# Therapeutic potential of medicinal plant sprouts: emerging opportunities and challenges in phytochemistry

**DOI:** 10.1007/s00425-025-04869-w

**Published:** 2025-11-05

**Authors:** Dominika Melegova, Andrea Babelova, Michal Selc

**Affiliations:** 1 Department of Nanobiology, Cancer Research Institute, Biomedical Research Center, Slovak Academy of Sciences, Bratislava, Slovakia; 2 Centre for Advanced Material Application, Slovak Academy of Sciences, Bratislava, Slovakia

**Keywords:** Seed, Sprouts, Germination, Phytochemicals, Secondary metabolites, Therapeutic potential

## Abstract

**Main conclusion:**

Germination is a developmental stage in which plants accumulate higher levels of bioactive metabolites. However, this potential remains largely unexplored in medicinal herbs and therapeutic applications.

**Abstract:**

Sprouting represents a brief yet metabolically dynamic phase in plant development, during which dormant seeds initiate enzymatic activation and begin synthesizing a range of bioactive compounds. Compared to dry seeds or mature plants, sprouts often contain higher levels of phenolics, flavonoids, vitamins, and other secondary metabolites, enhancing their nutritional and pharmacological value. While edible sprouts from food crops have been extensively studied, sprouts derived from medicinal plants remain largely overlooked. This is surprising given their natural richness in pharmacologically relevant phytochemicals. This perspective draws primarily on original experimental studies that investigated phytochemical and physiological changes during sprouting of medicinal plants. These studies were identified through searches in scientific databases using keywords related to medicinal plants, sprouts, and bioactive compounds. We summarize existing studies on species such as *Trigonella foenum-graecum*, *Nigella sativa*, *Silybum marianum*, *Arctium lappa*, *Trifolium pratense*, and *Glycyrrhiza uralensis*, and identify other promising candidates with high germination potential but uncharacterized phytochemical profiles at the sprout stage. Environmental variables, such as light quality, seed priming, or nutrient supplementation, can further modulate phytochemical composition during germination. We also discuss practical limitations, including low biomass yield, microbial safety concerns, and lack of standardized protocols. Overall, sprouts offer a responsive, scalable, and experimentally tractable model for exploring and optimizing phytochemical expression in medicinal plants. We propose that this early developmental window offers overlooked opportunities for phytopharmacology, functional foods, and natural product discovery.

## Introduction

Sprouts represent the earliest developmental stage of a plant, in which a dormant seed transforms into a metabolically active organism capable of autonomous growth. This phase is marked by rapid transformations: hydrolytic enzymes are activated, storage macromolecules are broken down, and secondary metabolism is stimulated, leading to increased accumulation of phenolics, flavonoids, and antioxidants that enhance the nutritional and health-promoting properties of sprouts (Benincasa et al. 2019; Majid et al. 2023). These transformations not only increase compound levels but may also improve their extractability and bioavailability, further contributing to the enhanced functional properties of sprouts. As a result, sprouts often exhibit a substantial increase in biological activity and nutritional value compared to ungerminated seeds (Liu et al. 2022).

In recent years, there has been growing scientific and consumer interest in sprouts as functional foods and sources of bioactive compounds, with most attention directed toward edible sprouts from food crops (Waliat et al. 2023). In contrast, medicinal plant sprouts remain largely outside the scope of mainstream research, even though they may offer equally, if not more, promising properties in the biomedical field. To date, no clinical trials have specifically evaluated medicinal sprouts, and their proposed health benefits rely only on preclinical findings and extrapolations from the traditional uses of mature plants. This gap likely reflects both the historical focus of pharmacognosy on mature plant parts and the practical challenges of sprouting, such as low biomass yield and microbial safety, which complicate systematic study.

This article explores the broader potential of sprouts as a platform for bioactive compound research and therapeutic innovation. In the sections that follow, we review the limited data on medicinal plant sprouts, discuss how their phytochemical content can be modulated, and outline the main challenges and opportunities that define this overlooked developmental stage.

## Sprouts from medicinal plants

While edible sprouts from food crops have been extensively studied and even commercialized, sprouts derived from medicinal plants remain a remarkably underexplored field. This is surprising given that medicinal plants are, by definition, rich in biologically active compounds, many of which are influenced by developmental stages and environmental factors, potentially including germination (El-Saadony et al. 2025). Medicinal plants have been used for centuries in various traditional systems of medicine due to their content of flavonoids, alkaloids, terpenes, saponins, and other specialized metabolites, typically extracted from roots, leaves, flowers, or seeds of mature plants (Petrovska 2012). However, very little is known about how these phytochemicals behave during the early sprout stage, whether they increase, decrease, or transform into novel metabolites with different biological effects. Despite this knowledge gap, a handful of studies have started to investigate germination in select medicinal species.

Fenugreek (*Trigonella foenum-graecum*), traditionally used as a demulcent, digestive aid, and for inflammatory and metabolic disorders, provides one of the clearest examples. Its sprouts exhibit enhanced antioxidant and antidiabetic activity compared to seeds, likely due to increased 4-hydroxyisoleucine and trigonelline content (Gonda et al. 2023; Çoban 2025). In addition, fenugreek sprout extract showed a stronger cytotoxic effect against HeLa and SiHa cervical cancer cell lines than seed extract, likely reflecting higher levels of bioactive content (Silva Comilo et al. 2025).

Black cumin (*Nigella sativa*) is a traditional remedy for respiratory, renal, hepatic, digestive, and cardiovascular disorders (Ahmad et al. 2013). Its sprouts show elevated phenolic content and preliminary anticancer activity. Thymol and thymoquinone, major bioactive compounds in sprout extract with known hepatoprotective and antioxidative properties, were reported to inhibit histone deacetylase in hepatocellular carcinoma. Thymoquinone also enhanced cytoprotective enzyme activity, which prevent lipid peroxidation supporting the antioxidative potential of black cumin sprouts (Algaissi et al. 2025).

Greater burdock (*Arctium lappa*) has been used in traditional for metabolic, renal, lung, skin, and digestive disorders (Grosu and Ichim 2020). It is rich in phenolic acids, lignans, notably in arctigenin and arctiine, and flavonoids. Burdock sprouts contain markedly higher level of arctigenin than seeds (13.7% vs. 0.1%) (Kravtsova and Khasanov 2011; Watanabe et al. 2023). Arctigenin from sprouts showed preventive effects against obesity in mice by reshaping the gut microbiota (Watanabe et al. 2023), and a clinical study reported reduced oxidized LDL and stabilized liver fibrosis markers in overweight adults consuming burdock sprout extract (Ito et al. 2021). These findings indicate potential benefits of burdock sprouts for metabolic health and liver function.

Red clover sprouts (*Trifolium pratense*) are rich in isoflavones such as formononetin and biochanin A. A clinical study reported reduction in fasting insulin, HbA1c, and glucose levels after daily consumption (Masuda et al. 2021), while animal experiments showed prevention of metabolic syndrome through improved lipid and glucose profiles (Yokoyama et al. 2020). Isoflavones from sprouts also act as phytoestrogens with affinity for estrogen receptor β, indicating potential benefits for menopausal symptom relief (Budryn et al. 2018). Overall, red clover sprouts may offer both metabolic and hormonal health benefits.

Milk thistle (*Silybum marianum*), traditionally used to treat liver and gallbladder disorders, shows remarkable phytochemical changes during germination. Polyphenol levels in sprouts can reach up to 640% higher than in mature plants, accompanied by enhanced antioxidant capacity under constant light. Furthermore, cultivation for 9–12 days at 75% visible light intensity significantly increases silymarin, the flavonolignan complex responsible for the plant’s hepatoprotective and antioxidant activity (Vaknin et al. 2008; Ropuszyńska-Robak et al. 2025).

Licorice (*Glycyrrhiza uralensis*), traditionally used for treating respiratory and other disorders (El-Saber Batiha et al. 2020), has shown promising effects at the sprout stage. Sprout extract, rich in bioactive compounds such as glycyrrhizin and liquiritigenin, significantly reduced intracellular lipids, triglycerides, and cholesterol, and inhibited H_2_O_2_-induced lipid peroxidation. In vivo, licorice sprout extract promoted healthy weight gain and enhanced antioxidant enzyme activities in plasma and liver tissues of rats (Park et al. 2024).

Taken together, these examples indicate that germination in medicinal plants often leads to the upregulation of phenolic and flavonoid biosynthesis, resulting in enhanced antioxidant and metabolic activities. While the specific compounds vary by species, the general trend points to sprouts as a developmental stage where bioactive potential is consistently amplified. To illustrate the current state of the field, Table 1 summarizes selected medicinal species that have been studied for sprouting research.

**Table 1:** Overview of selected medicinal plants traditionally used for various diseases and their bioactive compounds

Plant species	Common name	Traditional use	Bioactives in plant	Major phytochemical change in sprouts	References
*Arctium lappa*	Burdock	Renal, respiratory, dermatological, digestive	Phenolics (flavonoids, lignans, phenolic acids)	Arctigenin	Grosu and Ichim (2020), Ito et al. (2021), Watanabe et al. (2023)
*Astragalus membranaceus*	Mongolian milkvetch	Fatigue, loss of appetite, and dyspnea	Phenolics (flavonoids), polysaccharides	Formononetin, calycosin	Seo et al. (2024)
*Codonopsis lanceolata*	Deodeok	Asthma, cough, and psychoneurosis	Phenolics (flavonoids, phenolic acids, tannins), saponins	Total phenolic content, luteolin, lancemaside A	Choi et al. (2023), Kim et al. (2025)
*Glycyrrhiza uralensis*	Licorice	Digestive, respiratory, epilepsy,	Phenolics (flavonoids), saponins	Glycyrrhizin	El-Saber Batiha et al. (2020), Park et al. (2024)
*Nigella sativa*	Black cumin	Anti-inflammatory, digestive issues, respiratory issues, skin conditions	phenolics (flavonoids, phenolic acids), terpenoids, quinones	Thymol, thymoquinone	Tabassum and Ahmad (2018), Algaissi et al. (2025)
*Silybum marianum*	Milk thistle	Hepatoprotective	Phenolics (flavonolignans)	Silymarin	Vaknin et al. (2008), Ropuszyńska-Robak et al. (2025)
*Trifolium pratense*	Red clover	Respiratory, eczema, arthritis, gynecological	Phenolics (isoflavones)	Formononetin, biochanin A	Budryn et al. (2018), Yokoyama et al. (2020), Masuda et al. (2021)
*Trigonella foenum-graecum*	Fenugreek	Demulcent, digestive aid, and for inflammatory and metabolic disorders	Alkaloids, saponins, phenolics (flavonoids)	α-Tocopherol, trigonelline, 4-hydroxyisoleucine	Gonda et al. (2023), Çoban (2025), Silva Comilo et al. (2025)

## Bioactive compounds in sprouts

**Fig. 1:**
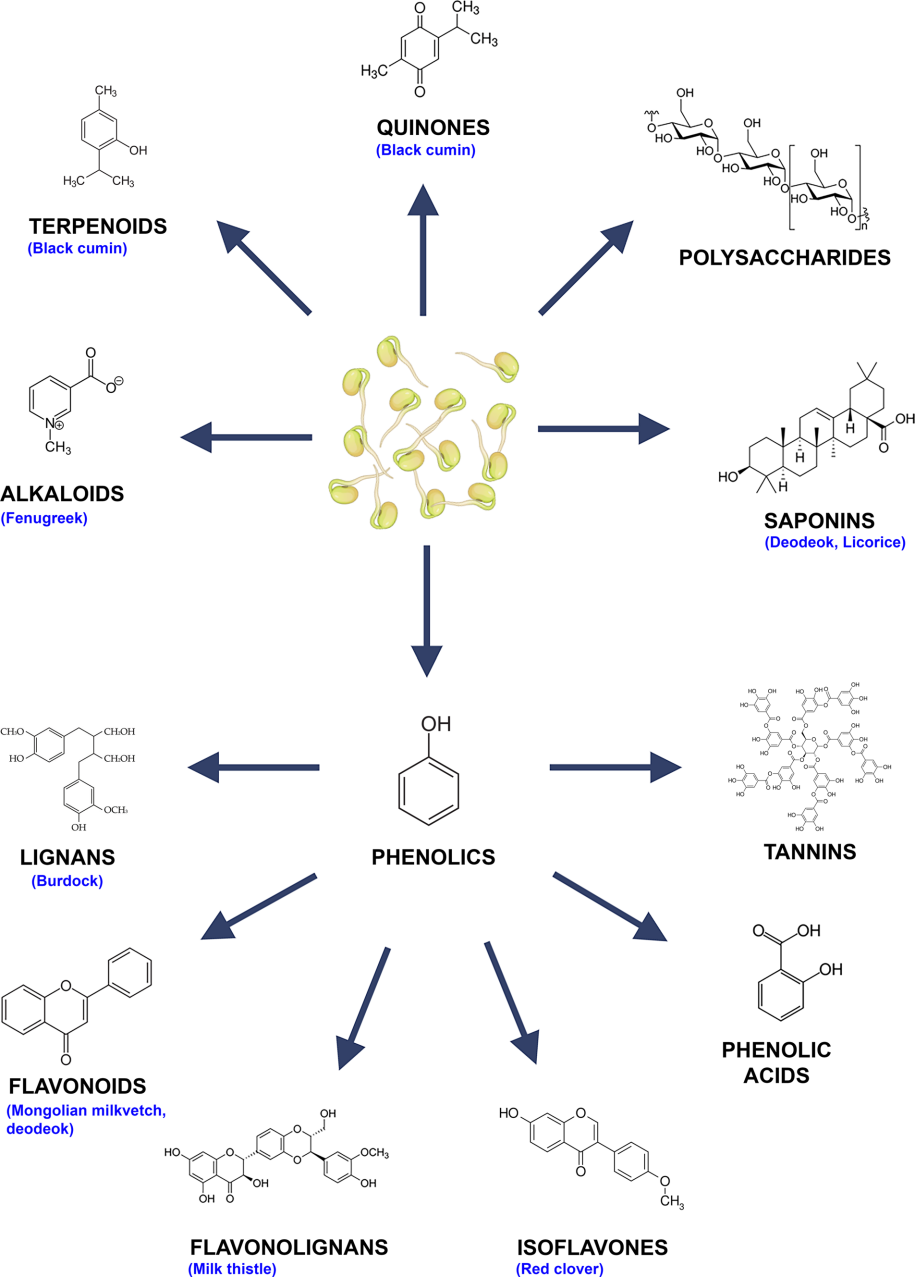
Major classes of bioactive compounds identified in medicinal plants. Sprouting is associated with dynamic changes in phytochemical composition, including alkaloids, terpenoids, quinones, polysaccharides, saponins and phenolics (lignans, flavonoids, flavonolignans, isoflavones, phenolic acid, and tannins). Medicinal plants in which these compound classes undergo the most pronounced changes during sprouting are shown in blue

Sprouting does not only represent the beginning of plant growth; this chemically and metabolically transformative phase activates complex enzymatic and physiological pathways which leads to dynamic changes in biochemical composition of the dormant seeds, including increased concentrations, diversity and bioavailability of phytochemicals such as flavonoids, polyphenols, vitamins, glucosinolates, and antioxidants (Afshari et al. 2024). The main classes of bioactive compounds identified in medicinal plants are summarized in Fig. 1. During these changes, degradation of antinutritional compounds occurs. Meanwhile, activation of secondary metabolic pathways is initiated, which leads to an increase in nutrient bioavailability and health-promoting phytochemicals. Several studies have demonstrated that the content of bioactive compounds is significantly higher, sometimes 10 to hundreds times, in sprouts than in ungerminated seeds and even in the mature plant, pointing towards enhanced anti-inflammatory, antioxidant, antidiabetic, and hypocholesterolemic potential (Ebert 2022; Afshari et al. 2024).

One of the most consistent observations across plant species is the increase in phenolic compounds during germination. A study by Liu et al. (2022) explored phytochemical profiles, phenolic metabolomics, and antioxidant capacity of 17 selected edible seeds and their sprouts. They found that sprouts had significantly higher total phenolic content and antioxidant activity compared to seeds. They have identified 316 phenolic metabolites between the sprouts and seeds, with 198 showing significant differences. Of these, 146 metabolites were up-regulated, while 52 were down-regulated in the sprouts. Furthermore, several metabolic pathways, including flavone and flavonol biosynthesis, phenylpropanoid biosynthesis, phenylalanine metabolism, and secondary metabolite production, in the sprouts were increased, which indicates that germination triggers the activation of defense and bioactive compound synthesis pathways. Finally, higher total phenolic content and phenolic metabolite abundance were consistently associated with increased antioxidant capacity in sprouts, indicating a close relationship between phenolic enrichment and antioxidant activity (Liu et al. 2022). These findings raise the possibility that sprouting in medicinal plants could lead to even more pronounced shifts in phytochemical profiles. Studies have shown that, in several evaluated species, sprouts exhibit higher phenolic content compared to seeds. This phytochemical composition can be further modulated by external factors such as light quality, temperature, nutrient availability, and even sound waves.

A study by Kim et al. (2021) demonstrated that exposing 1–6 day-old sprouts of various edible species to specific sound frequencies (0.25, 0.8, 1.0, and 1.5 kHz) increased flavonoid content by 25–88% compared to controls, depending on plant species and treatment regimen. The observed effect may be explained by sound functioning as a mechanical stress elicitor, which activates defense-related signaling cascades and thereby promotes the synthesis of secondary metabolites, including flavonoids. The increased flavonoid content has been correlated with up-regulated expression of biosynthetic genes such as PAL, C4H, 4CL, CHS, CHI, F3H, DFR, and ANR. Furthermore, the treated sprouts have also exhibited significantly higher antioxidant activity, consistent with their elevated flavonoid content (Kim et al. 2021).

Specific LED light spectra have been demonstrated to enhance isoflavonoid accumulation in Mongolian milkvetch sprouts (*Astragalus membranaceus*). White LED light exposure significantly increased formononetin accumulation, while red LED light promoted higher calycosin levels. This study demonstrates that tailored light spectra can help optimize the production of desired medicinal compounds in sprouts (Seo et al. 2024).

Seed priming with the nutrient priming agent potassium nitrate (KNO₃) was found to enhance germination and increase sprout fresh weight in linseed. KNO₃-primed sprouts have also exhibited elevated carbohydrate and amino acid content along with higher nitrogen assimilation enzyme activity. Levels of chlorophyll a/b and carotenoids were similarly increased in sprouts primed with ascorbic acid (AsA). However, AsA priming resulted in strong antimicrobial activities and a significant increase in total polyphenols, flavonoids, ascorbic acid, tocopherols, and antioxidant enzyme activities (Zrig et al. 2023).

The nutritional quality of wheat and millet (*Panicum miliaceum*, L.) sprouts has been shown to vary depending on germination temperature and duration. Ceccaroni et al. demonstrated that in wheat sprouts kept at lower temperature (15 °C), the synthesis and availability of bioaccessible freephenolic compounds increased, while bound phenolic compounds remained stable or decreased. After 3 days, the concentration of total polyphenols increased by 79.8% in the Gentil Roso (GR) genotype and by 65.8% in wheat mix (WM). At 20 °C, WM exhibited a significant increase of 81.5% in bound polyphenols, whereas GR showed only a mild increase of 25.2% after 3 days. On the other hand, the opposite trend was observed in millet sprouts, with both free and bound phenolic compounds being significantly increased at 20 °C. Total phenolics also rose in both wheat genotypes with ferulic acid representing 63–73% of total phenolic content. Free phenolic increased more consistently at 15 °C, while bound phenolic acids showed higher levels at 20 °C. Regarding sugars, the levels of saccharose, glucose and maltose in wheat sprouts increased significantly at 20 °C during the first two days. In millet sprouts, sugar accumulation was strongly temperature-dependent, with levels at 20 °C being approximately three times higher than at 15 °C. These findings indicate that nutritional quality of sprouts can be modulated by adjusting germination conditions, depending on the desired nutritional outcome (Tiansawang et al. 2016; Ceccaroni et al. 2020).

Beyond intrinsic plant metabolism, associated microbes can also act as modulators of bioactive compound formation. Rhizospheric and endophytic microorganisms are known to elicit secondary metabolic pathways in plants, enhancing the accumulation of phenolics, flavonoids, alkaloids, and terpenoids (Lv et al. 2024). Similarly, seed-borne endophytes have been shown to influence host physiology already at the germination stage (Hu et al. 2025). Although direct evidence from medicinal sprouts is still lacking, these findings suggest that microbial interactions could represent an overlooked factor shaping phytochemical profiles during sprouting.

In summary, the sprouting stage offers a brief yet highly responsive window for phytochemical enrichment. Through simple adjustments to environmental factors, such as light, temperature, or elicitor treatments, it is possible to direct the biosynthetic capacity of sprouts towards specific directions. Understanding and harnessing this chemical plasticity could facilitate new opportunities not only in nutrition and agriculture but also in pharmacognosy and natural product discovery.

## Limitations of sprouts

Despite their many advantages, sprouts are not without limitations, both as experimental systems and as sources of bioactive compounds. Their simplicity and speed come with trade-offs that must be considered when using them in research or for practical applications. One of the most fundamental limitations is the small amount of plant material that sprouts produce. A typical sprouting cycle yields only a few grams of biomass per seed batch, which can restrict their use in downstream analyses that require larger quantities of extract, purified compounds, or biological assays. For research purposes, this means careful planning of replication and harvest schedules; for commercial use, it presents a challenge in scaling production of phytochemical-rich sprouts (Narina et al. 2012; Chethan et al. 2021). Potential solutions include controlled-environment cultivation, such as vertical farming or bioreactor systems, which may help overcome scaling challenges (Verdú-Navarro et al. 2023; Sowmya et al. 2024). Moreover, low yield is less restrictive for modern mass spectrometry methods. Recent advances such as LC-HRMS, ion mobility, spatial metabolomics, and even quantitative single-cell MS enable comprehensive profiling from microgram or even single-cell quantities. For instance, quantitative single-cell MS has been used to identify and quantify natural products across multiple metabolite classes directly from individual plant cells, demonstrating that sprouts, despite their low biomass, remain tractable systems for deep metabolic exploration (Vu et al. 2024).

Sprouts are chemically dynamic, with metabolite profiles that can shift within hours depending on species and environmental conditions. While this offers opportunities for fine-tuning, it also adds a layer of complexity: reproducibility requires precise control over timing, environment, and harvest technique. Inconsistent timing can easily lead to contradictory or non-reproducible results (Ramakrishna et al. 2019).

Due to their high-water content, delicate tissue, and intense respiration, sprouts degrade rapidly after harvest. They can lose up to 27.3% of fresh weight just 3 days after harvest and even when refrigerated their shelf life is no longer than 5 days. This short shelf life complicates logistics for both experimental work and product development. Unless processed immediately or stored under ideal conditions, the quality and phytochemical content of sprouts can deteriorate within hours to days (Shomodder et al. 2022).

Sprouting requires warm, humid conditions, exactly the same conditions that favor microbial growth. This makes sprouts particularly vulnerable to contamination by pathogens such as *Salmonella enterica*, *Escherichia coli*, and *Listeria monocytogenes*, especially when using untreated or poorly sanitized seeds. For this reason, sprout cultivation, especially in food or medical research contexts, requires careful hygiene, sometimes involving sterilized growth chambers, autoclaved water, or antimicrobial treatments. These precautions increase labor and technical requirements, and may be prohibitive in low-resource settings (Gilbert et al. 2023; Xavier et al. 2025). Such microbial contamination is not only a safety concern, but may also unpredictably alter the phytochemical composition of sprouts (Young Kim et al. 2022; Fracchia et al. 2024). In response, non-thermal decontamination strategies such as cold plasma and UV-C treatment are being investigated as alternative approaches to improve microbial safety without compromising phytochemical quality (Fan et al. 2017; Waskow et al. 2018).

Unlike mature medicinal plants, for which pharmacopoeial methods exist, sprouts lack standardization. There are few established protocols for germinating edible seeds, assessing their phytochemical profiles, or comparing bioactivity across species and growth stages but they still vary among studies. Even basic parameters such as optimal sprouting time, light exposure, or harvest point can vary greatly between studies. This lack of methodological consistency makes it difficult to compare findings and build a coherent body of knowledge (Benincasa et al. 2019).

In most jurisdictions, sprouts are treated as foods rather than herbal products or medicines. This creates ambiguity when exploring their therapeutic use, especially for sprouts derived from traditionally “medicinal” herbs. Whether they fall under food safety laws, herbal medicine regulations, or neither, depend on the juridical system of each country, potentially complicating product development and translational research (Rojas et al. 2022).

Together, these limitations do not negate the value of sprouts, but they do highlight the need for careful experimental design and realistic expectations. As research in this area grows, it will be important to develop standard protocols, optimize cultivation parameters, and explore preservation or processing strategies that retain phytochemical integrity. For now, sprouts remain a promising but logistically delicate system, ideal for proof-of-concept studies, rapid screening, and metabolic exploration, but not yet ready for every application.

## Conclusion

Sprouts represent a brief but highly dynamic phase of plant life, a developmental stage where dormant seeds transform into metabolically active organisms capable of autonomous growth. Positioned between seeds and mature plants, they are simple enough to handle experimentally yet already biochemically diverse. This stage is not only important from a botanical perspective, but increasingly relevant to pharmacology, nutrition, and biotechnology.

Despite their simplicity, sprouts offer a powerful platform for discovering and enhancing bioactive compounds. Their fast growth, metabolic responsiveness, and low input requirements make them ideal for screening phytochemical responses to light, stress, or elicitor treatments. In some cases, sprouting can dramatically increase the levels of compounds with antioxidant, anti-inflammatory, or even anticancer activity. This has been well documented in several food crops, and initial evidence suggests that medicinal plants may respond similarly, if not more dramatically.

Yet the potential of medicinal sprouts remains largely untapped. Most herbal pharmacology focuses on mature plant parts, leaves, roots, flowers, while overlooking the developmental stages that may offer unique or amplified phytochemical profiles. Given the long history of medicinal plant use, it is somewhat surprising that sprouting has not become a more common strategy to enhance or diversify their therapeutic potential.

To be clear, sprouts are not a perfect substitute for mature plants or extracted compounds. Their low biomass, short shelf life, and susceptibility to microbial contamination pose real challenges. However, in contexts where small amounts of material are sufficient, such as early-phase bioactivity testing, compound discovery, or nutraceutical formulation, they may offer unique advantages. Their responsiveness to environmental factors also makes them an appealing model for studying plant metabolism under controlled conditions.

Perhaps most intriguingly, sprouts may be viewed as a developmental “sweet spot”: still simple enough to manipulate easily, but already biochemically active and diverse. They may also serve as a bridge to microgreens, which develop a more robust tissue structure while retaining much of the metabolic intensity of early growth stages.

In conclusion, future research should first and foremost establish the scientific value of medicinal sprouts through systematic phytochemical profiling and biological evaluation. Once their potential is more firmly demonstrated, more complex approaches—such as optimization under controlled-environment cultivation or scaling through vertical farming and bioreactor systems—will become meaningful. The encouraging results from the first experimental studies strongly suggest that such investment will be worthwhile, as sprouts may serve not only as functional foods but also as potential sources of novel therapeutic agents and as flexible systems for metabolic exploration that could open new frontiers in phytotherapy and natural product science.

## Declarations

### Data availability

No new data were generated or analyzed in this study.
